# The Role of Osteopontin and Its Gene on Glucocorticoid Response in Myasthenia Gravis

**DOI:** 10.3389/fneur.2017.00230

**Published:** 2017-05-31

**Authors:** Yanchen Xie, Hai-Feng Li, Liang Sun, Linda L. Kusner, Shuhui Wang, Yunxiao Meng, Xu Zhang, Yu Hong, Xiang Gao, Yao Li, Henry J. Kaminski

**Affiliations:** ^1^Department of Neurology, The George Washington University, Washington, DC, United States; ^2^Department of Neurology, Beijing Friendship Hospital, Capital Medical University, Beijing, China; ^3^Department of Neurology, Qilu Hospital of Shandong University, Jinan, China; ^4^The Key Laboratory of Geriatrics, Beijing Hospital, Beijing Institute of Geriatrics, Ministry of Health, Beijing, China; ^5^Department of Pharmacology, The George Washington University, Washington, DC, United States; ^6^Department of Physiology, The George Washington University, Washington, DC, United States; ^7^Department of Pathology, Peking Union Medical College Hospital, Chinese Academy of Medical Science, Beijing, China; ^8^Department of Neurology, Affiliated Hospital of Qingdao University, Qingdao, China

**Keywords:** myasthenia gravis, glucocorticoid, osteopontin, secreted phosphoprotein 1, quantitative myasthenia gravis score

## Abstract

Biomarkers that assess treatment response for patients with the autoimmune disorder, myasthenia gravis (MG), have not been evaluated to a significant extent. We hypothesized the pro-inflammatory cytokine, osteopontin (OPN), may be associated with variability of response to glucocorticoids (GCs) in patients with MG. A cohort of 250 MG patients treated with standardized protocol of GCs was recruited, and plasma OPN and polymorphisms of its gene, secreted phosphoprotein 1 (*SPP1*), were evaluated. Mean OPN levels were higher in patients compared to healthy controls. Carriers of rs11728697*T allele (allele definition: one of two or more alternative forms of a gene) were more frequent in the poorly GC responsive group compared to the GC responsive group indicating an association of rs11728697*T allele with GC non-responsiveness. One risk haplotype (AGTACT) was identified associated with GC non-responsiveness compared with GC responsive MG group. Genetic variations of *SPP1* were found associated with the response to GC among MG patients.

## Introduction

Myasthenia gravis (MG) is a chronic autoimmune disorder caused by antibodies directed against postsynaptic proteins, primarily the skeletal muscle nicotinic acetylcholine receptor (AChR). Oral glucocorticoids (GCs) are the primary therapy (1, 2), but response rates to GCs are highly variable among studies ranging from 5 to 30% (3–7). No clinical or biological markers exist that predict GC responsiveness. Genetic polymorphisms have been identified that are associated with therapeutic response to GCs in other autoimmune and inflammatory conditions (8), and we recently identified a single nucleotide polymorphism (SNP) in the GC receptor gene as an independent factor associated with short-term GC responsiveness among patients with MG (9).

Osteopontin (OPN) is a pro-inflammatory cytokine, and increased circulating levels have been associated with inflammatory muscle diseases and muscular dystrophy (10–13) as well as the onset and progression of Crohn’s disease, myocarditis, uveitis, idiopathic retroperitoneal fibrosis, and rheumatoid arthritis (14–16). Furthermore, *SPP1* gene polymorphisms have been identified as being associated with GC responsiveness in boys with Duchenne muscular dystrophy (13); however, a larger validation study published after we began our work did not confirm the association (17). We hypothesized that plasma OPN may be a marker of treatment responsiveness and that genetic variations (polymorphisms) in the secreted phosphoprotein 1 (*SPP1*) gene, which encodes OPN, are associated with differences in GC responsiveness of patients with MG.

We assessed the relationship among clinical characteristics and plasma OPN level of MG patients. Further, we evaluated the relationship between SNPs of *SPP1* gene and response to a standardized 3-month GC treatment protocol with prospectively collected outcome data. As our primary outcome assessment, we used the quantitative MG score (QMGS), which is a validated scale and was recommended by the MG Foundation of America as the primary clinical outcome measure for clinical trials (18). Several studies using the QMGS have shown that a change of 3 or more points to be clinically meaningful (19–21).

## Subjects and Methods

### Study Population

We assessed the same cohort as in our separate association study of *GR* gene polymorphisms and response to GCs (9). Three hundred forty-two consecutively identified MG patients who had not received immunosuppressive agents were recruited and followed from Beijing Friendship Hospital, Capital Medical University, and Affiliated Hospital of Qingdao University. The diagnosis of MG was based on a typical clinical history of variable weakness involving ocular, bulbar, limb, or a combination of muscle groups. Fatigable weakness was evident on physical examination. Alternative diagnoses, such as central nervous system disorders, myopathies, and motor neuron disorders were excluded. A positive result in at least one of three was required: (1) increased serum level of anti-acetylcholine receptor antibody (AChRAb); (2) decremental response to low frequency repetitive nerve stimulation; or (3) positive response to the neostigmine test. Muscle specific kinase patients were excluded.

**Figure 1:**
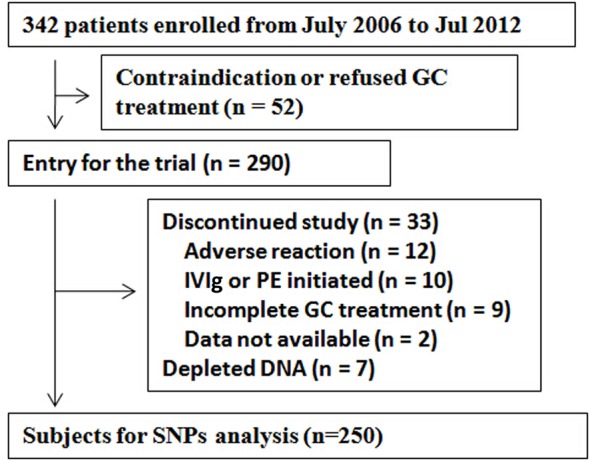
**Enrollment profile**. GC, glucocorticoid; IVIg, intravenous immune globulin; SNP, single nucleotide polymorphism.

Fifty-two patients were excluded because of a contraindication to GC therapy or refusal to receive GC treatment. The GC treatment was initiated with 0.75–1 mg/kg/day of prednisone or equivalent methylprednisolone. The dosage of GCs was tapered gradually when definite improvement was appreciated or was maintained for 3 months. Patients who had received plasma exchange, intravenous immunoglobulin or immunosuppressants during the study period were excluded. Patients who were excluded for other causes are described in Figure 1. DNA samples from seven patients were depleted from use in our previous study (9). Two hundred fifty patient samples underwent *SPP1* genotyping. A subset of 74 MG patients and 50 healthy controls underwent evaluation for plasma for OPN levels. Patients were stratified into subgroups by gender, age of onset (22), clinical presentation at disease onset, AChRAb status, presence of thymoma, disease duration before treatment, and QMGS before treatment (Table 1). Patients were followed monthly for 3 months after treatment initiation and QMGS determined by a physician trained in its performance (18). The control group consisted of 474 healthy individuals age-matched to the study population and seen during the study period at each participating institution. All study participants were northern Han Chinese and non-consanguineous. Change of QMGS was used as a primary efficacy measurement. Improvement of 3 or greater points of the QMGS or a QMGS becoming 0 identified a patient as being responsive to GCs (21, 23). The study was approved by ethical committees of the hospitals, and all participants provided written informed consent.

**Table 1:** **Comparison of the demographic and clinical characteristics of patients between responsive and non-responsive patients with MG**.

Variables	Responsive (*n* = 231)	Non-responsive (*n* = 19)	*p* Value
Age of onset (years)	44.08 ± 16.38	42.74 ± 19.39	0.734
Gender			0.403
Male	87	9	
Female	144	10	
Thymoma			0.056
Absence	176	14	
Presence	55	10	
Anti-AChR antibody			0.786
Negative	62	4	
Positive	161	14	
Involved muscles at disease onset			0.581
Ocular muscle	160	12	
Generalized muscle	71	7	
Thymectomy			0.280
No	203	15	
Yes	28	4	
**Disease duration before usage of GCs**
Within 6 months	159	9	0.055
After 6 months	72	10	
QMGS before treatment (median, months)	6	7	0.992

*MG, myasthenia gravis; AChR, acetylcholine receptor; GC, glucocorticoid; QMGS, quantitative myasthenia gravis score*.

### Enzyme-Linked Immunosorbent Assay (ELISA)

Blood samples were collected in ethylenediaminetetraacetic acid from patients prior to the initiation of GC or any other immunotherapy. Plasma was isolated and stored at −80°C until evaluation. The concentration of OPN was determined by the quantitative sandwich ELISA using the Quantikine kit (R&D Systems, Minneapolis, MN, USA). Antibodies against AChR (AChRAb) was detected by using ELISA kit (RSR Limited, Cardiff, UK) (24). The testing was performed according to the instructions of the kit. The results were expressed as inhibition rate of AChR binding, calculated according to the formula in the instructions: Inhibition rate (%) = 100 × (test sample absorbance/negative control absorbance).

### SNP Selection and Genotyping

Twelve SNPs (variations in single base pairs in a DNA sequence) were selected based on previous reports and information from NCBI dbSNP and HapMap database [CHB database, HapMap phase version 3, release 27 (2009, February)], in an attempt to cover the majority of the *SPP1* gene region by linkage disequilibrium (LD). Among the 12 SNPs, 1 tag SNP (rs2853749) was selected using the HapMap database with the software as previously described, and 11 SNPs (rs2728127, rs2853744, rs11730582, rs11439060, rs11728697, rs6840362, rs4754, rs1126616, rs4660, rs1126772, and rs9138) were previously reported (25–33). Ten of these have functional potential (rs2728127, rs2853744, rs11730582, rs11439060 in the 5′ near gene; rs11728697, rs4754, rs1126616, rs4660 in coding region; rs1126772, rs9138 in 3′-untranslated region). One SNP has been previously investigated (rs6840362), which showed a significant difference allele distribution in European American patients with SLE (30). The location and function of the SNPs are shown in Table 2.

**Table 2:** **Twelve SNPs in healthy control, GC responsive, and GC non-responsive groups**.

SNP (major/minor)	Function	Genetic models	Control^c^ (*n* = 474)	Responsive^c^ (*n* = 231)	Non-responsive^c^ (*n* = 19)	HWE^‡^	*p* Value^a^	OR^b^ (95% CI)
rs2728127	5′ near	ALLELIC	584/364	290/170	26/12	0.41	0.508	0.787 (0.387–1.601)

A/G	Gene	GENO	169/246/59	88/114/28	9/8/2		0.74	
		DOM	169/305	88/142	9/10		0.44	0.69 (0.27–1.76)
		REC	415/59	202/28	17/2		0.83	0.85 (0.19–3.87)

rs2853744	5′ near	ALLELIC	578/370	290/170	26/12	0.12	0.508	0.787 (0.387–1.601)

G/T	Gene	GENO	168/242/64	88/114/28	9/8/2		0.74	
		DOM	168/306	88/142	9/10		0.44	0.69 (0.27–1.76)
		REC	410/64	202/28	17/2		0.83	0.85 (0.19–3.87)

rs11730582	5′ near	ALLELIC	613/335	310/152	25/13	0.48	0.701	1.146 (0.571–2.301)

T/C	Gene	GENO	202/209/63	104/102/25	6/13/0		0.035	
		DOM	202/272	104/127	6/13		0.25	1.77 (0.65–4.83)
		REC	411/63	206/25	19/0		0.232	0.92 (0.88–0.95)

rs11439060	5′ near	ALLELIC	579/369	288/174	26/12	0.15	0.456	0.764 (0.376–1.553)

–/G	Gene	GENO	169/241/64	88/112/31	9/8/2		0.73	
		DOM	169/305	88/143	9/10		0.43	0.68 (0.27–1.75)
		REC	410/64	200/31	17/2		0.71	0.76 (0.17–3.45)

rs2853749	Intron 1	ALLELIC	579/369	285/173	26/12	0.1	0.448	0.760 (0.374–1.546)

C/T		GENO	168/243/63	85/115/29	9/8/2		0.68	
		DOM	168/306	85/144	9/10		0.38	0.66 (0.26–1.68)
		REC	411/63	200/29	17/2		0.78	0.81 (0.18–3.70)

rs11728697	Intron 3	ALLELIC	556/392	275/187	19/19	0.78	0.252	1.471 (0.758–2.852)

C/T		GENO	161/234/79	83/109/39	2/15/2		0.018*	
		DOM	161/313	83/148	2/17		0.014*	4.77 (1.07–21.14)
		REC	395/79	192/39	17/2		0.45	0.58 (0.13–2.61)

rs6840362	Intron 3	ALLELIC	909/39	447/15	36/2	1	0.378	1.656 (0.364–7.524)

C/T		GENO	435/39/0	216/15/0	17/2/0		0.53	
		DOM	435/39	216/15	17/2		0.63	1.69 (0.36–8.03)
		REC	474/0	231/0	19/0		NA	NA

rs4754	Exon 6	ALLELIC	703/245	335/127	29/9	0.55	0.612	0.819 (0.377–1.777)

C/T		GENO	263/177/34	119/97/15	11/7/1		0.86	
		DOM	263/211	119/112	11/8		0.59	0.77 (0.30–1.99)
		REC	440/34	216/15	18/1		0.83	0.80 (0.10–6.41)

rs1126616	Exon 7	ALLELIC	703/245	335/127	29/9	0.55	0.612	0.819 (0.377–1.777)

T/C		GENO	263/177/34	120/95/16	11/7/1		0.87	
		DOM	263/211	120/111	11/8		0.62	0.79 (0.31–2.03)
		REC	440/34	215/16	18/1		0.77	0.75 (0.09–5.96)

rs4660	Exon 7	ALLELIC	948/0	480/0	20/0	NA	NA	NA

G/A		GENO	948/0/0	240/0/0	10/0/0		NA	
		DOM	948/0	240/0	10/0		NA	NA
		REC	948/0	240/0	10/0		NA	NA

rs1126772	3′ UTR	ALLELIC	685/263	340/122	29/9	0.42	0.714	0.865 (0.398–1.879)

A/G		GENO	251/183/40	125/90/16	10/9/0		0.24	
		DOM	251/223	125/106	10/9		0.9	1.06 (0.42–2.71)
		REC	434/40	215/16	19/0		0.619	0.92 (0.88–0.95)

rs9138	3′ UTR	ALLELIC	702/246	334/128	29/9	0.47	0.593	0.81 (0.373–1.758)

		GENO	263/176/35	119/96/16	11/7/1		0.86	
		DOM	263/211	119/112	11/8		0.59	0.77 (0.30–1.99)
		REC	439/35	215/16	18/1		0.77	0.75 (0.09–5.96)

*^‡^p Value for Hardy–Weinberg equilibrium test among healthy controls*.

*^a,b^Comparison between GC responsive MG with GC non-responsive MG*.

*^c^Major homozygotes/heterozygotes/minor homozygotes*.

*ALLELIC, allelic test; GENO, genotypic test; DOM, dominant gene action test; REC, recessive gene action test; HWE, Hardy–Weinberg equilibrium; NA, not applicable; GC, glucocorticoids; MG, myasthenia gravis; SNP, single nucleotide polymorphism*.

The significant results are highlighted in bold. *p < 0.05.

Single nucleotide polymorphism genotyping was performed using a custom-designed SNPscan^TM^ Kit (Genesky Biotechnologies Inc., Shanghai, China). For quality assurance, 3.87% (28/724) of the total samples were randomly repeated. Concordance for duplicate samples was 100% for all assays.

### Statistical Analysis

Statistical analysis was performed with SPSS Statistics 13 (SPSS Corporation, Chicago, IL, USA), SHEsis software (Bio-X Life Science Research Center, Shanghai, China), Haploview 4.2 software, and GraphPad Prism 5 (GraphPad Software, Inc., La Jolla, CA, USA). A two-side comparison with *p* < 0.05 was considered statistically significant. The database, constructed using SQL server, contains data pertaining to the SNPs, clinical features and treatment, and clinical follow-up of patients.

The normality of the data was tested using the method of Kolmogorov and Smirnov. Continuous variables were presented as mean ± SD or median, 25th and 75th percentiles; categorical variables were presented as a percentage. Differences between groups were analyzed with independent sampled *t* test or the Mann–Whitney *U*-test for continuous variables and by chi-square test or Fisher exact test for categorical variables. The association between the GC efficacy and MG phenotype and *SPP1* genotypes was examined by multivariate regression analysis. In this pilot study, we did not make a correction for multiple comparisons. The Haploview 4.2 software was used to calculate pairwise LD of SNPs and construct haplotype blocks. Haplotypes are defined as genetic variations that are inherited together. Haplotype frequencies were estimated with Partition–Ligation–Combination–Subdivision Expectation Maximization algorithm implemented in SHEsis software.

### Functional Annotation and Expression Quantitative Trait Locus (eQTL) Analysis

Functional annotations of SNPs were investigated using RegulomeDB, a database which provides assessment of whether SNPs are located in known or predicted regulatory elements, including regions of DNase I hypersensitivity, binding sites for transcription factors (TFs), and promoter regions that regulate transcription (34).

With the aim of exploring the molecular basis of the observed associations, eQTLs analysis was performed by using published cell-specific eQTL dataset (35).

## Results

### Patient Characteristics

The study cohort consisted of 250 patients of which 154 (61.6%) were women (Table 1). The onset age ranged from 15 to 80 years, mean 43.98 ± 16.59 years. The disease duration prior to GC therapy ranged from 0.2 to 48 months with a median duration of 4 months (interquartile range of 2–11 months). Among 474 healthy controls, 238 were men, and 236 are women with an age range of 14–78 and median age of 45 years.

#### Response to GCs

Quantitative MG score ranged from 1 to 35 (median QMGS was 6, interquartile ranged from 4 to 11) at study onset, and after 3 months of treatment, a significant reduction in QMGS was observed ranging from 0 to 29 points (median QMGS was 1, interquartile ranged from 0 to 3, *p* < 0.0001). The change in QMGS ranged from −2 to 18 (median QMGS was 5, interquartile ranged from 3 to 8).

Two hundred thirty-one patients (92.4%) were considered GC responsive, and 19 (7.6%) were considered GC non-responsive. Clinical characteristics of the patients are summarized in Table 1. No differences were found between a poor response to GC therapy with age of onset, gender, presence of thymoma, AChR antibody status, muscle group involvement at disease onset, thymectomy, disease duration before GC treatment, or QMGS before treatment (*p* = 0.73, 0.41, 0.78, 0.79, 0.59, 0.28, 0.06, and 0.99, respectively) (Table 1).

### OPN Concentrations in MG Patients and Healthy Controls

**Figure 2:**
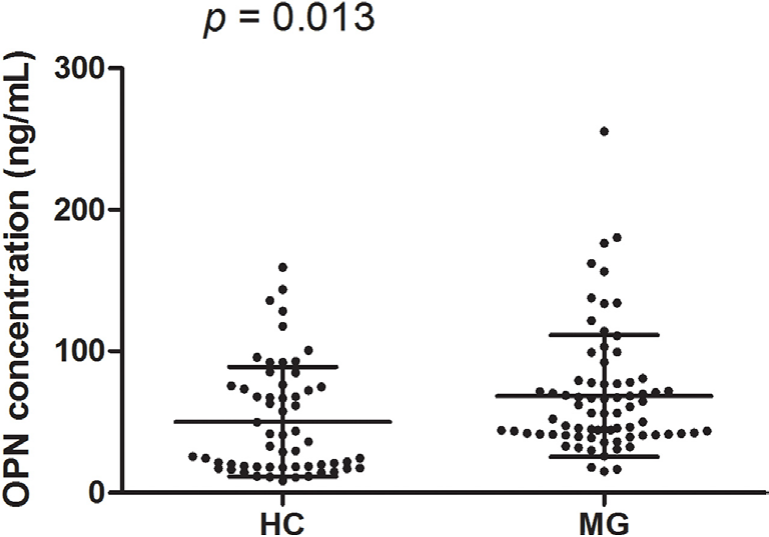
**Osteopontin (OPN) levels among myasthenia gravis (MG) patients and healthy controls**. Mean OPN levels were higher in MG patients (68.33 ± 43.03 ng/ml) compared to healthy controls (50.19 ± 38.74 ng/ml; *p* = 0.013).

**Figure 3:**
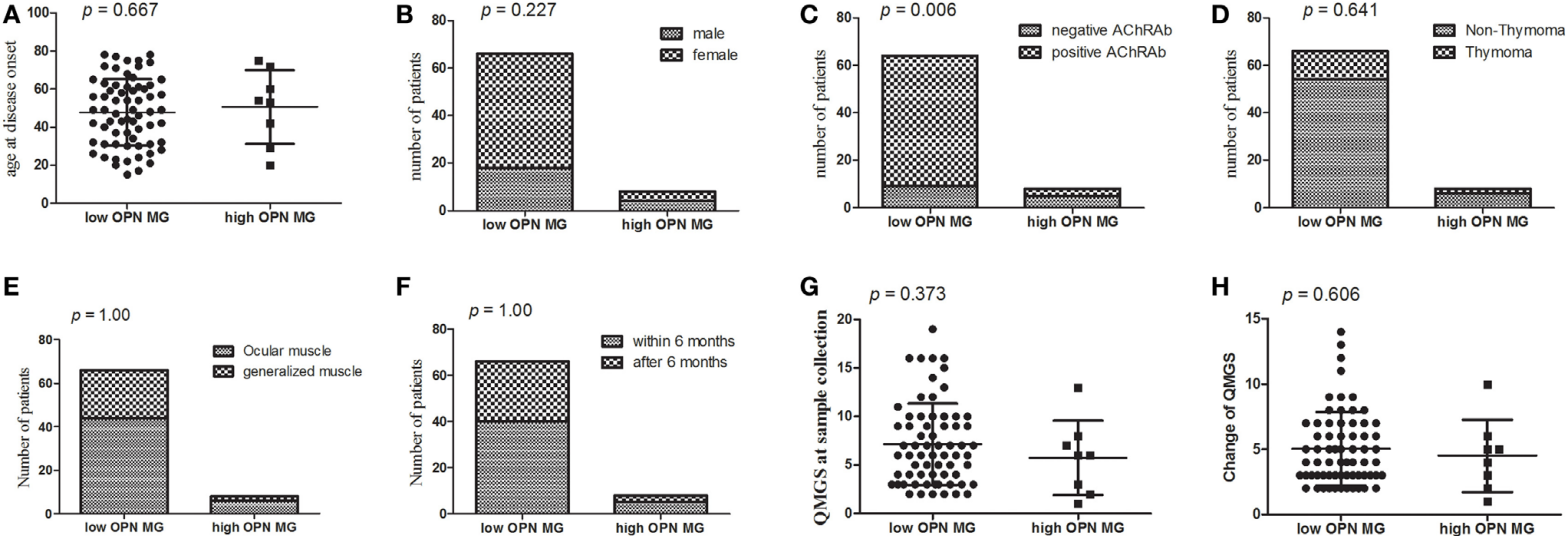
**Clinical features of myasthenia gravis (MG) patients with the greatest osteopontin (OPN) levels**. The MG patients with the highest levels of OPN had lower percentage of positive acetylcholine receptor (AChR) antibody (37.5 vs 85.94%, *p* = 0.006, Figure 3C). No difference in age of disease onset, gender, presence of thymoma, involved muscle at disease onset, duration before glucocorticoid (GC) treatment, quantitative MG score (QMGS) at the sample collection, and change of QMGS after 3 months GC treatment (*p* = 0.667, 0.227, 0.641, 1.00, 1.00, 0.373, 0.606, respectively, Figures 3A,B,D–H).

Prior to initiation of immunotherapy, mean OPN plasma levels were greater among MG patients (68.33 ± 43.03 ng/ml) compared to healthy controls (50.19 ± 38.74 ng/ml; *p* = 0.013; Figure 2). We performed a subgroup analysis of the patients with the highest OPN levels (mean + 2 SD of controls; 127.67 ng/ml in the study), which identified 8 patients, and compared them to the remaining 66 patients (Figure 3). We found that the MG patients with the highest levels of OPN had lower percentage of positive AChRAb (37.5 vs 85.94%, *p* = 0.006, Figure 3C). No difference in onset age, gender, presence of thymoma, involved muscle group at disease onset, duration before GCs treatment, QMGS at the sample collection, and change of QMGS after 3 months GC treatment (*p* = 0.667, 0.227, 0.641, 1.00, 1.00, 0.373, 0.606, respectively, Figures 3A,B,D–H) was found between the two subgroups.

### Association between *SPP1* Gene Variation and Response to GC

The success rates of genotyping the 12 SNPs among MG patients and healthy controls were greater than 99.7%. None of the participants had the rs4660 polymorphism. The genotyping data of 12 SNPs of the healthy controls did not deviate from the Hardy–Weinberg equilibrium (HWE, *p* = 0.1–1, Table 2). General characteristics of 12 SNPs in the *SPP1* gene of patients are shown in Table 2. The distribution of genotypes in the GC sensitive and insensitive groups was consistent with the Hardy–Weinberg equilibrium (Table 2).

The rs11728697 T carriers (C/T + T/T genotypes) were more frequent in the GC non-responsive group compared to the GC responsive group (89.5 vs 64.1%; dominant model: *p* = 0.014; OR = 4.77, 95% CI = 1.07–21.14), indicating association with a poor response to GCs. No statistically significant differences were observed for the remaining SNPs (Table 2). No difference in the frequency of the rs11728697 T carriers was observed between MG patients and healthy control groups (66 vs 66%, dominant model, *p* = 0.99).

The association between *SPP1* gene variation and responses to GCs was further examined by multivariate logistic regression analysis, with GC non-response as the dependent variable, and with onset age, gender, involved muscles at disease onset, AChRAb, presence of thymoma, disease duration before GC treatment, and rs11728697 as independent variables. The rs11728697 T carrier was found as an independent factor for GC non-responsiveness (*p* = 0.019, OR = 4.76, 95% CI = 1.03–21.99).

### *SPP1* Haplotypes and GC Efficacy in MG Patients

**Figure 4:**
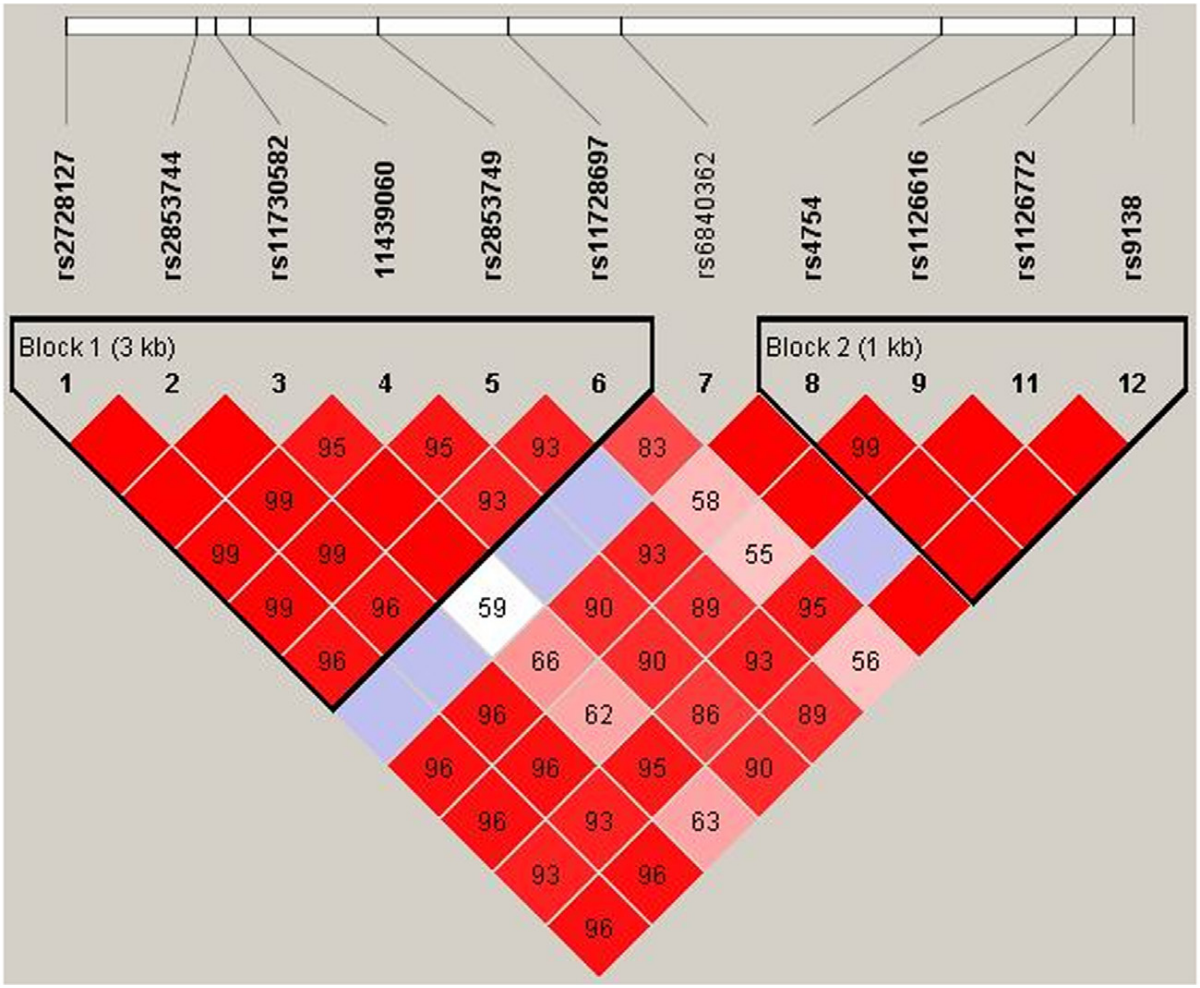
**Haplotype block of *SPP1* variants**. Generated by Haploview (version 4.2). Dark red, strong linkage disequilibrium (LD); light red, weak LD.

**Figure 5:**
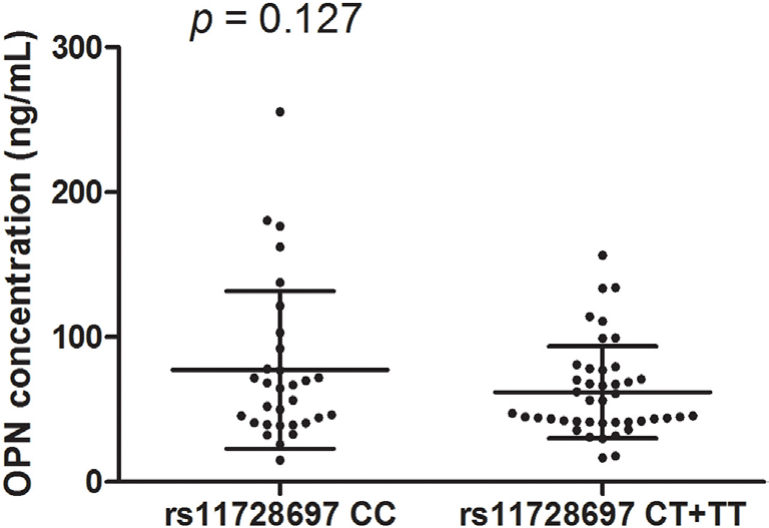
**Osteopontin (OPN) concentrations in subgroups with rs11728697 genotypes**. No difference in OPN concentrations was found between rs11728697 CC (77.34 ± 54.36 ng/ml) and rs11728697 CT + TT (61.84 ± 31.68 ng/ml).

The LD test among 12 SNPs in the *SPP1* gene is shown in Figure 4. According to Haploview, the haplotype block structure of the *SPP1* gene consists of two blocks. The first block ranges from rs2728127 to rs11728697 (rs2728127, rs2853744, rs11730582, rs11439060, rs2853749, and rs11728697; *D*′ ranging from 0.93 to 1.0), the second from rs4754 to rs9138 (rs4754, rs1126616, rs1126772, and rs9138; *D*′ ranging from 0.99 to 1.0). A total of four common haplotypes were identified across the first block, ranging in frequency from 35.5 to 7.3% in all patients (GTTGTC: 35.5%, AGCACT: 32.6%, AGTACC: 21.5%, AGTACT: 7.3%). One risk haplotype (AGTACT) in the first block was identified (OR = 2.61, 95% CI = 1.09–6.73, *p* = 0.041) in the GC non-responsive MG group compared with GC responsive MG group (15.8 vs 6.5%) (Table 3). Three common haplotypes were identified across the second block, ranging in frequency from 46.4 to 27.0% in total patient (CTAC: 46.4%, TCAA: 27.0%, CTGC: 26.2%). No risk haplotype was found across the second block. There was no difference of OPN concentration between MG patients with rs11728697 CC (77.34 ± 54.36 ng/ml) and those with rs11728697 CT + TT (61.84 ± 31.68 ng/ml; *p* = 0.127) (Figure 5).

**Table 3:** **Haplotype analysis of the *SPP1* gene variants between glucocorticoids responsive and non-responsive subjects**.

ID	Haplotype	Frequency		χ*^2^*	*p*	OR (95% CI)
		Responsive	Non-responsive			
1	GTTGTC	0.355	0.316	0.414	0.520	0.792 (0.389–1.613)
2	AGCACT	0.326	0.342	0.003	0.955	1.021 (0.507–2.053)
3	AGTACC	0.219	0.184	0.372	0.542	0.768 (0.328–1.797)
4	AGTACT	0.065	0.158	4.185	0.041*	2.606 (1.009–6.729)

*Haplotypes constructed by rs2728127, rs2853744, rs11730582, rs11439060, rs2853749, and rs11728697*.

The significant results are highlighted in bold. *p < 0.05.

### RegulomeDB Analysis of rs11728697

According to the functional annotation information from the RegulomeDB scoring, rs11728697 was identified as score 1d (http://www.regulomedb.org/snp/chr4/88898940) with evidence for eQTL, TF binding, a matched TF binding motif, and location within DNase-sensitive site. rs11728697 is within the region of the binding site of TF regulatory factor X 3 that was detected by Chip-Seq analysis in K562 cell line (36). rs11728697 was found to be linked with rs12502049 (*D*′ = 0.958 and *R*^2^ = 0.724), which was identified as score 1f with evidence for mapping to a predicted TF binding site and/or within a DNase I sensitivity peak and correlating with gene expression. rs12502049 is known to regulate the expression of *SPP1* gene that was detected by eQTL analysis in a lymphoblastoid cell line (37).

## Discussion

We identified an SNP in the *SPP1* gene (rs11728697), which was associated a poor response to GC treatment among patients with MG. One risk haplotype (AGTACT) containing a mutation at this SNP was identified among the GC non-responsive patients compared with the GC responsive patients. The carrier of the variant does not differ between MG patients and the controls, indicating the association of rs11728697 T with GC response is not determined by its involvement in pathogenesis of MG but associates with GC treatment response. Presently, there is no evidence of a direct interaction between OPN and GC response pathways; however, OPN influences T and B cell function, which provides indirect pathways that could be associated with GC treatment responsiveness (38). Further, rs11728697 is in a location of the *SPP1* gene that may bind transcriptional factors offering a mechanism for GC effect.

Previous studies of genetic variations of response to GC therapy in autoimmune or inflammatory disorders are limited. Investigation of GC responsiveness in SLE identified SNP associations in the GC receptor gene (39). We also identified an SNP in the GC receptor gene that was associated with a poor treatment response among patients with MG (9). A study of Duchenne muscular dystrophy found an rs28357094 polymorphism, which lies in the gene promoter of *SPP1*, to be associated with GC response. In this study, we aimed to explore whether polymorphisms in the gene of an immuno-modulating protein was associated with GC responsiveness in MG.

There is no accepted definition of treatment response for MG therapeutics, which is a limitation of our investigation. As more precise clinical and biological definitions of treatment response are identified, genetic and biomarker studies may identify more robust associations. The QMGS is a validated scale and was recommended by the MG Foundation of America Task Force as the primary clinical outcome measure (19). Several studies have indicated that an improvement of 3 or more points on the QMG scale is clinically meaningful (19–21). Pascuzzi and colleagues treated 116 patients with similar regimens of 60–80 mg daily of prednisone and with prolonged follow-up, 5% were described as unresponsive to treatment (3). Our result is consistent with the findings of this investigation. Others have found higher rates of limited treatment response using less strict definitions from ours. However, their study had a longer observational length, which would indicate that treatment failure may have been contaminated by patients developing GC complications. A retrospective study over 2 years of observation found 13% of patients were unchanged or worse, and in a long-term study of 104 patients, 13 percent had no improvement in MG manifestations with treatment (6, 7). Further, an observational study without a standardized treatment protocol found about one-third of patients have significant disability despite 6 months of treatment, which usually included prednisone (40).

We found that the mean plasma concentration of OPN was significantly increased in MG patients compared with controls, which is consistent with its role as a pro-inflammatory cytokine and is consistent with its elevation in other autoimmune and inflammatory diseases (38). We did not appreciate an association of OPN levels with any clinical parameters or genotypes of this SNP. There are several potential explanations. Circulating cytokine levels have disease-related and diurnal variations, which may not have been accounted for by this study. The investigation may not have been able to distinguish disease-specific variations due to a lack of statistical power. From the present investigation whether circulating OPN levels or mutations in *SPP1* may be predictive of treatment response cannot be determined. *SPP1* expression is elevated in muscle of animals with passive transfer MG (41). OPN has been found to be involved in progression of endogenous autoreactive germinal centers leading to enhanced antinuclear antibody production in an animal of lupus (42), which points to the multiple mechanisms that are involved in autoantibody production. No difference in the proportion of rs11728697 T carrier was found in association with the plasma level of OPN. This likely relates to several potential factors impacting circulating levels of OPN, including other genetic associations with GC response.

In summary, increased mean plasma levels of OPN are found among MG patients, but these had no relationship to patient demographics or treatment response. We found a mutation at rs11728697 and two haplotypes in the *SPP1* gene to be associated with poor response to GC treatment among MG patients. In particular, subgroup analyses did not find associations with clinical characteristics of patients, but conclusions are limited because of small sample sizes. A larger validation study will be required to confirm these observations. For MG and other autoimmune disorders, it is critical to move toward identification of markers that predict treatment response with the intent of providing individual treatment (43).

## Ethics Statement

The study was approved by ethical committees of Beijing Friendship Hospital, Capital Medical University, and Affiliated Hospital of Qingdao University, and all participants provided informed consent.

## Author Contributions

YX, H-FL, and HK conceptualized and designed the study, interpreted the data, and wrote the manuscript. LS designed the genotyping experiment and performed bioinformatics analysis. LK contributed to discussion. H-FL, SW, XZ, XG, and YL diagnosed and treated patients for this study. YX, LS, and YH performed statistical analysis. YM maintained the database and determined the pathologic diagnosis for the study.

## Conflict of Interest Statement

YX received research support from Beijing Natural Science Foundation. H-FL received research support from National Natural Science Foundation of China and Shandong Provincial Natural Science Foundation. YM received research support from National Natural Science Foundation of China. HK receives grant support from the Myasthenia Gravis Foundation of Illinois and the Muscular Dystrophy Association, fees for serving on data and safety monitoring boards from Genentech and Novartis, consulting fees from Alnylam Pharmaceuticals, UCB, Rubius Therapeutics, RA Pharmaceuticals, and Momenta Pharmaceuticals, receiving study medication from Genentech, and holding a patent related to technology to focus a complement inhibitor on the neuromuscular junction for the treatment of myasthenia gravis (U.S. patent no. 8,961,981). LK receives grant support from the Muscular Dystrophy Association. All other authors report no disclosures.

## Abbreviations

AChR, acetylcholine receptor; GC, glucocorticoid; ELISA, enzyme-linked immunosorbent assay; eQTLs, expression quantitative trait loci; IVIG, intravenous immunoglobulin; LD, linkage disequilibrium; MG, myasthenia gravis; OPN, osteopontin; PE, plasma exchange; QMGS, quantitative MG score; SNP, single nucleotide polymorphism; *SPP1*, secreted phosphoprotein 1.
